# The Relationship between Toll-like Receptors and *Helicobacter pylori*-Related Gastropathies: Still a Controversial Topic

**DOI:** 10.1155/2019/8197048

**Published:** 2019-02-04

**Authors:** Lorena Elena Meliț, Cristina Oana Mărginean, Cristian Dan Mărginean, Maria Oana Mărginean

**Affiliations:** ^1^Department of Pediatrics, University of Medicine, Pharmacy, Sciences and Technology Tîrgu Mures, Gheorghe Marinescu Street No. 38, 540136, Romania; ^2^University of Medicine, Pharmacy, Sciences and Technology of Tîrgu Mures, Gheorghe Marinescu Street No. 38, 540136, Romania

## Abstract

Innate immunity represents the first barrier against bacterial invasion. Toll-like receptors (TLRs) belong to the large family of pattern recognition receptors (PRRs), and their activation leads to the induction of inflammatory cytokines, chemokines, antigen-presenting molecules, and costimulatory molecules. Recent studies have focused on identifying the association between TLRs and *Helicobacter pylori*- (*H. pylori*-) related diseases. Therefore, this minireview focuses on assessing the role of these TLRs in the development of *H. pylori*-related gastropathies. Both TLR2 and TLR were found to be involved in *H. pylori* LPS recognition, with contradictory results most likely due to both the inability to obtain pure LPS in experimental studies and the heterogeneity of the bacterial LPS. In addition, *TLR2* was found to be the most extensively expressed gene among all the TLRs in gastric tumors. High levels of TLR4 were also associated with a higher risk of gastric cancer. TLR5 was initially associated with the recognition of *H. pylori* flagellin, but it seems that this bacterium has developed mechanisms to escape this recognition representing an important factor involved in the persistence of this infection and subsequent carcinogenesis. TLR9, the only TLR with both anti- and proinflammatory roles, was involved in the recognition of *H. pylori* DNA. The dichotomous role of TLR9, promoting or suppressing the infection, depends on the gastric environment. Recently, TLR7 and TLR8 were shown to recognize purified *H. pylori* RNA, thereby inducing proinflammatory cytokines. *TLR1* and *TLR10* gene polymorphisms were associated with a higher risk for gastric cancer in *H. pylori*-infected individuals. Different gene polymorphisms of these TLRs were found to be associated with gastric cancer depending mostly on ethnicity. Further studies are required in order to develop preventive and therapeutic strategies against *H. pylori* infections based on the functions of TLRs.

## 1. Introduction

*Helicobacter pylori* (*H. pylori*), a Gram-negative, microaerophilic, and spiral-shaped bacterium, infects the human gastric mucosa at an early age, colonizes the mucosal gel layer, and leads to chronic inflammation in the stomach [1–3]. This bacterium is present in the gastric mucosa of up to 50% of the world's population and is responsible for different conditions, such as chronic gastritis, peptic ulcers, gastric cancer, lymphomas of the gastric-associated lymphoid tissue, and gastric adenocarcinoma [4]. Nevertheless, gastropathies can also be caused by other factors and conditions, such as drugs, portal hypertension, bile reflux, uremia, Henoch-Schönlein purpura, and radiation [5, 6]. *H. pylori* was considered as a class I carcinogen by the World Health Organization (WHO), with gastric cancer being identified as the third leading cause of cancer-associated deaths worldwide [7, 8]. Individuals suffering from *H. pylori* infection were found to have a sixfold higher risk of developing gastric cancer and up to a 50% higher risk for all gastric cancers as compared to uninfected individuals [9, 10]. Carcinogenicity of *H. pylori* is regulated by 3 factors, namely, bacterial virulence constituents, host genetic susceptibility, and environmental factors [11]. The eradication of *H. pylori* infection in symptomatic individuals has been unsuccessful in recent years due to a constant increase in the antimicrobial resistance [12]. Among European individuals, resistance rates against metronidazole and clarithromycin have reached 34.9% and 17.5%, respectively, and strains that are more resistant to levofloxacin (14.1%), which was used as a rescue therapy for this infection, have also emerged [13]. This may be due to the fact that *H. pylori* has developed the ability to regulate T-cell responses [14]. Moreover, gastric biopsies from gastritis patients infected with *H. pylori* that failed to respond to a proper eradication regimen revealed that some strains of this bacterium, such as those with an altered lipopolysaccharide structure or those which inhibit cathepsin X, are able to mediate resistance against such drugs [15].

The immune system has an essential role in controlling infections, and both innate and adaptive immunity contribute to this process [14]. The innate immunity is considered as the first line of defense against pathogens [16]. Therefore, epithelial cells of the gastric mucosa are considered as the first line of innate immunity against an *H. pylori* infection [17]. The pathogen recognition receptors (PRRs) are known to recognize different bacterial molecular patterns, playing a major role in the stimulation of adaptive immunity [18]. The crosstalk between innate and adaptive immunity is critical for the successful eradication of different bacteria. Mammalian toll-like receptors (TLRs) belong to the large family of PRRs, and the activation of their signaling pathways results in the induction of inflammatory cytokines, chemokines, antigen-presenting molecules, and costimulatory molecules [19]. Multiple studies have focused on the identification and elucidation of such TLRs, and 10 types of TLRs in humans have been identified [20]. These receptors express the pattern of type I transmembrane proteins, which involves a leucine-rich repeat-containing ectodomain, a transmembrane region, and an intracellular domain, which is involved in the activation of downstream signaling pathways. Some of these TLRs, TLR1, 2, 4, 5, and 6, recognize different membrane components like lipids, lipoproteins, and proteins, binding their ligands on the cell surface, while others, including TLR3, 7, 8, and 9, play a major role in the recognition of microbial nucleic acids, which are identified in intracellular vesicles [18, 21–26]. The expression and activation of different TLRs in the gastric mucosa have been widely investigated by recent studies, but their precise role and functions are not yet fully understood. Additionally, due to the recently discovered increase in antimicrobial resistance, novel therapies involving innate immunity are required.

This review aims at identifying and characterizing the role of TLRs in *H. pylori*-related gastric pathologies.

### 1.1. TLR2 and *H. pylori*

TLR2 has been shown to recognize bacterial lipopolysaccharide (LPS), acting as a primary barrier against *H. pylori* [21, 27]. Its expression results in the activation of different transcription factors, such as nuclear factor-*κ*B, stimulating the production and secretion of proinflammatory cytokines, such as interleukin- (IL-) 1*β*, IL-2, IL-6, IL-8, and IL-12 [28, 29]. An interaction between TLR2 and TLR4 has also been identified. This interaction consists of an unraveled subsequent expression of TLR4 as a result of the activation of TLR2, eventually leading to metaplasia, dysplasia, and adenocarcinoma [30, 31]. Together with TLR4, TLR2 is one of the best characterized TLRs. Besides recognizing LPS, TLR2 is also involved in the recognition of other bacterial components, such as lipoproteins or peptidoglycan [32]. In addition, it seems that TLR2 recognizes bacterial LPS with a different structure compared to those recognized by TLR4 [32]. A study performed on cell lines proved that *H. pylori* LPS acts as a classic TLR2 ligand by involving key TLR-signaling components [29]. This bacterial LPS also stimulates a mild chemokine expression in TLR2-expressing epithelial cells [29]. Nevertheless, the reports whether TLR2 is responsible for the recognition of *H. pylori* LPS remain contradictory. Multiple studies have shown that the recognition of this LPS by TLR2 results in the activation of nuclear factor-*κ*B, suggesting that the downregulation of TLR2 leads to the inhibition of cytokines mediated by *H. pylori* LPS [29, 33–36]. On the contrary, other studies have found TLR4 to be involved in the recognition of *H. pylori* LPS [17, 37–40], most likely based on its proven interaction with TLR2. In addition, as we have previously mentioned, TLR4 might be involved in the recognition of LPS having a different structure than that recognized by TLR2. Nevertheless, a recent study, performed on cell lines, stated that the *H. pylori* LPS induced a discrete pattern of chemokine expression only in TLR2-expressing cells and not in TLR4-expressing cells; this effect was mediated through the interaction between the LPS and TLR2 [29]. Nevertheless, when assessing these experimental studies, performed on a cultured cell line, we must take into account that it is very difficult to obtain pure LPS and to create the exact same conditions as in vivo. Besides these studies performed on cultured cell lines, TLR2 expression was found to be increased in gastric biopsy samples obtained from patients who tested positive for *H. pylori* infection [37, 41–43].

In addition to *H. pylori* LPS recognition, it was shown that TLR2 also triggers a Th1 immune response by binding to *H. pylori* neutrophil-activating protein [44]. TLR2 is also involved in the pathogenesis of gastric cancer worldwide. Different ethnic studies performed on gastric biopsy samples identified that genetic polymorphism in *TLR2* is associated with an increased risk of gastric cancer, with variations between different geographic areas and populations [45–47]. Contrariwise, certain *TLR2* polymorphisms, such as *TLR2 c. -196* to *-174 del* carriers (ins/del + del/del) were shown to be significantly associated with a decreased risk of gastric cancer in Chinese [45]. On the other hand, other studies performed in Japanese failed to identify any association between this polymorphism and gastric malignancies, suggesting that ethnic differences may modify the *H. pylori*-related carcinogenesis [48]. Moreover, the *TLR2* functional polymorphisms found in Caucasians, Pro631His, Arg677Trp, and Arg753Gln, differ from those identified in Asiatic people, *TLR2-688G>T*, *-264G>C*, and *c.2242ACA>del* [45, 49–51]. As in the case of gastric biopsy samples, the risk of gastric cancer due to peptic ulcers differs among individuals who harbor different *TLR2* gene polymorphisms [52], depending also on ethnic features as mentioned above. In addition, TLR2/MyD88 signaling is essential for both tumor cell stemness and gastric tumorigenesis being activated by the inflammatory microenvironment, as was proven by studies involving mouse models [53]. Therefore, the inhibition of this signaling pathway could represent a major preventive and therapeutic approach against gastric cancer [54]. In addition, in mice, TLR2 was found to be the most significantly upregulated gene among the TLRs in gastric tumor samples, its expression being predominantly identified in tumor epithelial cells [55]. Based on these findings, it is clear that TLR2 is involved in the recognition of *H. pylori* LPS and that there is an interaction between TLR2 and TLR4. There is strong evidence suggesting the role of TLR2 in the development of gastric cancer despite ethnic differences. Nevertheless, the differences regarding TLR polymorphisms between different people suggest that ethnicity is an essential factor that can modulate the response to *H. pylori* infection and its persistence. Therefore, despite limited literature, it is clear that TLR2 plays a major role in *H. pylori* recognition and gastric tumorigenesis.

### 1.2. TLR4 and *H. pylori*

TLR4 was the first TLR identified in humans, and similar to TLR2, it was associated with the recognition of bacterial LPS, an essential component of the outer membrane of Gram-negative bacteria [22]. Due to the wide structural diversity of the bacterial LPSs, as mentioned above, TLR4 is not able to recognize all types of LPSs. In order to stimulate an adequate response against an LPS and to induce secretion of inflammatory cytokines, TLR4 requires the aid of other molecules, such as MD-2, LPS-binding protein, and CD14 [16]. The initiation of the nuclear factor-*κ*B pathway due to *H. pylori* LPS recognition leads to the activation of the IL-8 pathway, which acts as a promoter of proinflammatory cytokines [56]. *H. pylori* heat shock protein 60 is another essential component of this bacteria that activates both TLR2 and TLR4, resulting not only in the increase of nuclear factor-*κ*B activity but also in the production of IL-8 in cultured gastric epithelial cells [57]. Nevertheless, it seems that the TLR4 expression remains unaltered in the gastric epithelium independently of the presence of *H. pylori* [58]. TLR4 is expressed at the apical and basolateral levels of the membrane compartment [58]. A more recent study performed on gastric biopsy specimens showed that expressions of both TLR4 and TLR2 are upregulated as a result of *H. pylori* infection in antral and gastric body areas [43]. The same study suggested that the expression of both the TLRs changed significantly only in the antral region compared to the control [43]. In order to perform their functions, both TLR4 and TLR2 need to be internalized into the cytoplasmic compartment. In a study conducted on cell lines infected with *H. pylori* strains from patients in whom the eradication of bacteria was unsuccessful, cathepsin X was shown to influence the internalization of both TLRs. Therefore, the inhibition of this cathepsin resulted in a suppression of their redistribution to the intracytoplasmic compartment, leading to a delay in the successful immune response against *H. pylori* [59]. The ability of *H. pylori* to trick the immune system has evolved progressively resulting in a persistent infection. Thus, this bacterium has acquired the ability to modify the lipid A core of its LPS, representing a possible ligand for the immune complex TLR4-MD2, in order to avoid TLR4 recognition [11]. In addition, *H. pylori* LPS was proven to have approximately 1000-fold less endotoxic properties in comparison to other bacteria due to this modification to the core [60, 61]. Therefore, it seems that the reduced functioning of TLR4 in individuals that carry hyporeactive polymorphisms in the gene for this TLR is associated with a reduced inflammatory response eventually leading to a persistent infection [62, 63]. T regulatory cells play an immunosuppressive role, and their numbers were shown to be higher in *H. pylori*-infected individuals [64]. A recent study performed on mice demonstrated an association between the TLR4 signaling pathway and T regulatory cells. The study showed that suppressing the TLR4 signaling pathway will eventually lead to an increase in T regulatory cells resulting in an exacerbation of *H. pylori* colonization [65]. However, the same study pointed out that inhibiting the CD25 expression could decrease T regulatory cells and, as a result, could suppress *H. pylori* colonization [65].

Even though certain TLR4 polymorphisms have been associated with a higher risk of developing premalignant conditions, such as hypochlorhydria and gastric atrophy [46], other studies, performed on bigger cohorts, failed to identify any such relationships [66, 67]. Similar to TLR2, the association between *TLR4* polymorphisms and the risk for carcinogenesis related to TLR4 most likely depends on ethnicity. Thus, a study performed on Chinese population suggested an association between TLR4 3725 G/C polymorphism and an increased risk of developing gastric cancer [68]. Nevertheless, other studies failed in proving this association in the Caucasian or Japanese populations [48, 69]. On the other hand, *TLR4Asp299Gly* gene polymorphism was found to increase the risk of developing gastric neoplasia in Caucasians [46] but not in Chinese patients [70, 71]. It seems that TLR4 is involved in the recognition of bacterial LPS structure that is different from the one recognized by TLR2. Moreover, it can be considered as a suppressor of *H. pylori* infection, and therefore, it could be valuable as a potential therapeutic regimen against this bacterium, especially in patients that fail to respond to the conventional therapeutic options.

### 1.3. TLR5 and *H. pylori*

TLR5 was identified as the receptor which recognizes bacterial flagellin, which is a protein component of the polymeric flagellar filament of certain Gram-positive and Gram-negative bacteria [23]. *H. pylori* contains approximately 5-7 flagella per cell, which provide motility and consist of two subunits, the major flagellin and the minor flagellin [72, 73]. The recognition of *H. pylori* by TLR5 is achieved through the p38 MAP kinase signaling pathway [24, 30, 74]. It is well documented that TLR5 is present on both primary gastric epithelial cells and gastric cell lines [24, 41]. Similarly, a recent study showed an association between a *TLR5* rs5744174 polymorphism and *H. pylori* infection [45]. However, a study performed on cell lines suggested that *H. pylori* flagellin cannot be recognized by TLR5 in case of a mutation in the conserved domain of the major subunit [75]. Moreover, the authors suggested that the major subunit of *H. pylori* flagella demonstrated low immunogenic features, as they were unable to activate TLR5 [75]. Thus, it was proven that Epsilonproteobacteria gastric pathogen *H. pylori* evolved its flagellin involving several amino acid changes within the D1 domain in order to escape TLR5 recognition, but it conserved its motility [75]. A more recent study performed on human embryonic kidney cell lines proved that the D0 domain also owns a role in TLR5 evasion along with specific amino acid residues that must be altered in the evolution of this Epsilonproteobacteria in order to accomplish its evasion [76]. Moreover, it seems that the TLR5 receptor does not necessarily require a specific ligand for its activation. Thus, it was proven that the autoactivation of the chimeric TLR5 relies on the specific binding of the linked flagellin to the TLR5 ectodomain similar to the binding of free flagellin to wild-type TLR5 [77]. These findings suggest the fact that TLR5 may become active without requiring a direct recognition of specific bugs or ligands. Additionally, Jerala et al. designed a fusion protein containing the *H. pylori* flagellin A, but they replaced the amino-terminal and carboxy-terminal segments with sequences of the flagellin from a bacterium that is able to activate TLR5 [78]. This invention was conceived for the preparation of the vaccine against bacteria whose flagellin is unable to activate TLR5. Therefore, *H. pylori* might have developed a defensive mechanism in order to escape from TLR5 detection leading to its persistence within the gastric niche and subsequent secretion of IL-8 [11].

The rs5744174 polymorphism was also found to be associated with an increased risk of gastric carcinogenesis in Chinese patients due to its potential ability that interferes with the ligand recognition, thereby allowing the persistence of *H. pylori* in the stomach [45]. As a result, in *H. pylori*-infected individuals that carry a “high-risk” genotype, the persistence of this infection will lead to an increased secretion of proinflammatory cytokines [79, 80]. These cytokines will create an inflammatory microenvironment and, eventually, will lead to the development of gastric malignancies [79, 80]. A more recent study, which included 1300 cases of gastric cancer and 1300 healthy controls from China, identified that two other polymorphisms in *TLR5*, namely, rs1640827 and rs17163737, were associated with *H. pylori* infection and, subsequently, with a high risk for gastric carcinoma [81]. Additionally, the authors encountered also a significant correlation between TLR5 rs5744174 and gastric cancer [81]. Contrariwise, in Caucasians, studies identified a functional polymorphism *TLR5* 392STOP [82], which was not identified in Chinese patients [45], underlining once more the importance of ethnic difference on the development of gastric malignancies. Therefore, it can be stated that different genetic variants of this receptor could influence the role of *H. pylori* in gastric carcinogenesis [81]. A study involving coculturing of monocytic THP-1 cells with wild-type *H. pylori* strains showed that virulent strains of *H. pylori* that carry the *cag* pathogenicity island (*cagPAI*) can be influenced by TLR2 and TLR5 [70]. This influence consists in shifting the proinflammatory pathways from *cagPAI*-dependent to *cagPAI*-independent, impacting the outcome of the diseases associated with *H. pylori* infection [83]. Based on all these data, it is clear that TLR5 plays an essential role in *H. pylori*-associated disorders, but further studies are needed in order to elucidate its precise function in the promotion or suppression of the bacteria. TLR5 recognizes *H. pylori* flagellin, even though studies performed on cell lines proved a potential escaping mechanism. We can also state that, most likely, the discordant findings can be due to different *TLR5* gene polymorphisms. Thus, there is a need to study the relationship between such gene polymorphisms and *H. pylori* infection, preferably in gastric biopsy specimens.

### 1.4. TLR9 and *H. pylori*

TLR9 is an endosome-transmembrane receptor involved in the recognition of DNA of invading microbes or damaged host cells [11, 25]. TLR9 can recognize pathogenic DNA via its structural components such as the saccharide backbone, in a sequence-independent manner [84–86]. The expression of this receptor differs in healthy individuals and individuals infected with *H. pylori*. TLR9 is located in the apical compartment of the gastric epithelial cells in healthy controls, while in individuals infected with *H. pylori*, it is located only in the basolateral compartment [58]. Rad et al. proved in a study performed on murine models that the *in vivo* recognition of *H. pylori* by TLR9 results in proinflammatory responses [87]. On the contrary, a more recent study performed on mice suggested that during the acute phase of *H. pylori* infection, TLR9 is able to promote anti-inflammatory signaling, thus acting as a suppressor for *H. pylori*-induced gastritis [88]. The microenvironment can also influence TLR9 to induce either proinflammatory or anti-inflammatory signaling resulting in a dichotomous role of this TLR in the presence of *H. pylori* [11]. Therefore, *H. pylori* can attenuate inflammatory response via TLR9 during the acute phase in order to establish persistent infection [11].

Nevertheless, the inflammatory microenvironment that contains cells without polarity can stimulate TLR9 to promote proinflammatory cascades and eventually result in the development of gastric cancer [30, 58, 89]. This hypothesis is additionally sustained by the fact that TLR9 is upregulated in cancer tissue [30, 58, 89]. Moreover, different *TLR9* SNPs have been associated with an increased risk of gastric malignancies [90–92]. A recent study performed by Varga et al. proved that the levels of TLR9 are significantly higher within the epithelial gastric cells of Colombian people from the high gastric cancer risk areas compared to those residing in the low-risk regions [26]. Additionally, the authors underlined that the *H. pylori* cancer-associated *cag* type IV secretion system (T4SS) is mandatory for the activation of TLR9 and that the *H. pylori* DNA is actively translocated by this system to engage this host receptor [26]. Ethnic differences were reported also in the case of this TLR. Thus, a study performed on 168 healthy Caucasian from the West of Scotland, first-degree relatives of gastric cancer patients, showed a significant association between a functional polymorphism *TLR9*-1237T>C and *H. pylori*-related premalignant gastric lesions [93]. Contrariwise, this polymorphism was rarely found in Chinese people [94].

Therefore, TLR9 plays a dichotomous role, being the only receptor that has both proinflammatory and anti-inflammatory roles. Its function is influenced by the microenvironment and especially by the presence of *H. pylori*. The discordant findings could be due to the fact that most of the studies were performed on animal models, and the human microenvironment is considerably different. Therefore, further studies on gastric biopsy specimens must be performed in order to identify the precise conditions that trigger either the proinflammatory or the anti-inflammatory function of TLR9.

### 1.5. Other TLRs and *H. pylori*

TLR7 and TLR8 have been shown to recognize purified *H. pylori* RNA which induces proinflammatory cytokines in an MyD88-dependent manner [87]. In addition, certain genetic polymorphisms of different proinflammatory cytokines, such as IL-6 190 C/T, IL-6 572 G/C, tumor necrosis factor alpha 308 G/A, and angiotensin-converting enzyme I/D, were associated with *H. pylori* infection in children [95]. Peng et al. showed that T regulatory cells expressed high levels of TLR8 [96]. Also, it seemed that TLR8 stimulation of T regulatory cells hinders their inhibitory action [96]. A recent study proved that *TLR1* rs4833095 and *TLR10* rs10004195 gene polymorphisms are associated with a higher risk of gastric cancer in *H. pylori*-infected individuals [97]. In addition, a recent genome-wide association study and meta-analysis showed that the TLR1-TLR6-TLR1 locus is associated with *H. pylori*-positive serology generated through TLR1 [98]. TLR1 and TLR6 could also be binding partners for TLR2, aiding in its ability to recognize different ligands [99]. More recent findings underlined that *H. pylori* seroprevalence could be attributed to TLR10 rather than TLR1, suggesting that TLR10 can be considered a functional receptor involved in the innate immune response triggered by *H. pylori* [100]. These TLRs are less studied than the ones mentioned above, but they seem to also play an important role in *H. pylori*-related gastropathies. Therefore, they should be the focus of future studies.

### 1.6. *H. pylori* and MALT Lymphomas

Most of the data reported in the literature focus on the interaction of *H. pylori* infection, TLRs, and gastric carcinomas. Despite the fact that studies regarding gastric lymphomas are scarce, the fact that *H. pylori* represents a definitive cure of low-grade gastric mucosa-associated lymphoid tissue (MALT) lymphoma was recognized worldwide and proven by multiple studies. Thus, a recent study underlined that the eradication of *H. pylori* resulted in complete remission in 84 of 99 patients diagnosed with this type of lymphoma, of which 94% presented long-term complete remission [101]. Moreover, persistence of *H. pylori* represents the most important risk factor for the recurrence of gastric low-grade MALT lymphoma requiring a proper eradication regiment accurate regular assessment of *H. pylori* infection [102]. On the other hand, *H. pylori* pathogenic features, like cagA, were also proven to influence lymphomagenesis by promoting the ability of lymphocytes to escape apoptosis [103].

Besides its well-documented carcinogenic role, it was recently proven that *H. pylori* might also be used as an anticarcinogenic agent. Thus, a study performed on mice showed that *H. pylori* neutrophil-activating protein (HP-NAP) might be useful in treating bladder cancer [104].

### 1.7. *H. pylori* Virulence Factors and TLRs

*H. pylori* infection may have a wide range of outcomes depending on both the pathogenic features of the bacteria and the individual's immune system. Therefore, in an ideal world, gastric epithelial cells are the first line of the innate immune response against this bacteria, and they own the ability to trigger multiple cell signaling cascades resulting in an outcome related to the host's inflammatory response. Human gastric epithelial cells were found to express both TLR2 and TLR4 leading to the contradictory results regarding *H. pylori* LPS recognition. As mentioned above, studies report that both TLR2 and TLR4 recognize this LPS. Thus, multiple hypotheses were postulated in order to explain these contradictory results, such as the differences in experimental studies and the difficulties related to the contamination during the LPS preparation or to the heterogeneity of its structures. Despite the difficulty of obtaining a pure LPS in experimental studies, a viable, final statement would be that both TLR2 and TLR4 are involved in *H. pylori* LPS recognition depending probably on both the host's immune system and *H. pylori* strain differences [105]. *H. pylori* flagellin is another bacterial component that owns a greater role in long-term bacterial persistence. Though it was proven to be recognized by TLR5, in vivo this bacterial flagellin is a less potent stimulator of the TLR5 signaling as compared to other Gram-negative bacteria [24, 73], providing *H. pylori* another potential mechanism to escape host immune response and persist in the human stomach [14, 24]. Another important component of *H. pylori* is the 60 kDa heat shock protein which is able to stimulate the IL-8 induction in the gastric epithelial cells, being related to gastric inflammation and MALT development [57]. This immune antigen was shown to be recognized by TLR2 [57, 106]. Peptidyl-prolyl cis-trans isomerase is a powerful compound secreted by *H. pylori*, and it owns the ability to induce gastric epithelial cells being recognized by TLR4 [107–109] resulting in subsequent promotion of Th1 immune responses [14]. *H. pylori* DNA is recognized by TLR9, while TLR8 and TLR7 are involved in the recognition of *H. pylori*-purified RNA [87]. As mentioned above, TLR9 is able to play a dichotomous role, promoting or suppressing *H. pylori* infection depending on the microenvironment. Besides all these *H. pylori*-virulent factors resulting in different escaping mechanisms that may promote the persistence of this infection within the stomach, hyporeactive polymorphisms of the TLRs, as mentioned above, are an additional risk factor for individuals who carry them to develop gastric malignancies.

## 2. Conclusions

Innate immunity plays an essential role in the promotion or suppression of *H. pylori* infection via TLRs. The discordant findings of different studies might suggest that each TLR can either promote or suppress an *H. pylori* infection, depending on other host-related factors. Moreover, the study design, the number of subjects included in the studies, or the methods used in each study can also influence the results. Taking into account all these facts, the findings of the studies performed on human gastric biopsies are probably the most accurate ones. Nevertheless, further studies are required in order to identify the exact role of each TLR in the development and pathogenesis of *H. pylori* infection and, subsequently, in gastric cancer.

## Abbreviations

*H. pylori*: *Helicobacter pylori*
HP-NAP: *H. pylori* neutrophil-activating protein
IL: Interleukin
LPS: Lipopolysaccharide
MALT: Mucosa-associated lymphoid tissue
PRRs: Pathogen recognition receptors
TLRs: Toll-like receptors.

## References

[1] Hofman P., Waidner B., Hofman V., Bereswill S., Brest P., Kist M. (2004). Pathogenesis of Helicobacter pylori infection. *Helicobacter*.

[2] McColl K. E. L. (2010). Clinical practice. Helicobacter pylori infection. *The New England Journal of Medicine*.

[3] Hessey S. J., Spencer J., Wyatt J. I. (1990). Bacterial adhesion and disease activity in Helicobacter associated chronic gastritis. *Gut*.

[4] Sijun H., Yong X. (2009). Helicobacter pylori vaccine: mucosal adjuvant & delivery systems. *The Indian Journal of Medical Research*.

[5] Dohil R., Hassal E. (2006). Chapter 25 - gastritis, gastropathy and ulcer disease. *Pediatric Gastrointestinal and Liver Disease (Third Edition)*.

[6] Meliţ L. E., Mărginean C. O., Mocanu S., Mărginean M. O. (2017). A rare case of iron-pill induced gastritis in a female teenager: a case report and a review of the literature. *Medicine*.

[7] McGuire S. (2016). World Cancer Report 2014. Geneva, Switzerland: World Health Organization, International Agency for Research on Cancer, WHO Press, 2015. *Advances in Nutrition*.

[8] Ferlay J., Soerjomataram I., Dikshit R. (2015). Cancer incidence and mortality worldwide: sources, methods and major patterns in GLOBOCAN 2012. *International Journal of Cancer*.

[9] Forman D., Newell D. G., Fullerton F. (1991). Association between infection with Helicobacter pylori and risk of gastric cancer: evidence from a prospective investigation. *BMJ*.

[10] Parsonnet J., Friedman G. D., Vandersteen D. P. (1991). Helicobacter pylori infection and the risk of gastric carcinoma. *The New England Journal of Medicine*.

[11] Varga M. G., Peek R. M., Tegtmeyer N., Backert S. (2017). DNA Transfer and Toll-like receptor modulation by *Helicobacter pylori*. *Current Topics in Microbiology and Immunology*.

[12] O’Connor A., Gisbert J. P., McNamara D., O’Morain C. (2011). Treatment of Helicobacter pylori infection 2011. *Helicobacter*.

[13] Megraud F., Coenen S., Versporten A. (2013). *Helicobacter pylori* resistance to antibiotics in Europe and its relationship to antibiotic consumption. *Gut*.

[14] Smith S. M. (2014). Role of toll-like receptors in *Helicobacter pylori* infection and immunity. *World Journal of Gastrointestinal Pathophysiology*.

[15] Skvarc M., Kopitar A. N., Kos J., Obermajer N., Tepes B. (2014). Differences in the antigens of *Helicobacter pylori* strains influence on the innate immune response in the in vitro experiments. *Mediators of Inflammation*.

[16] Kawai T., Akira S. (2009). The roles of TLRs, RLRs and NLRs in pathogen recognition. *International Immunology*.

[17] Mandell L., Moran A. P., Cocchiarella A. (2004). Intact gram-negative Helicobacter pylori, Helicobacter felis, and Helicobacter hepaticus bacteria activate innate immunity via toll-like receptor 2 but not toll-like receptor 4. *Infection and Immunity*.

[18] Kawai T., Akira S. (2011). Toll-like receptors and their crosstalk with other innate receptors in infection and immunity. *Immunity*.

[19] Chaudhary P. M., Ferguson C., Nguyen V. (1998). Cloning and characterization of two toll/interleukin-1 receptor-like genes TIL3 and TIL4: evidence for a multi-gene receptor family in humans. *Blood*.

[20] O’Neill L. A. J., Golenbock D., Bowie A. G. (2013). The history of toll-like receptors — redefining innate immunity. *Nature Reviews Immunology*.

[21] Castaño-Rodríguez N., Kaakoush N. O., Pardo A. L., Goh K.-L., Fock K. M., Mitchell H. M. (2014). Genetic polymorphisms in the toll-like receptor signalling pathway in Helicobacter pylori infection and related gastric cancer. *Human Immunology*.

[22] Poltorak A., He X., Smirnova I. (1998). Defective LPS signaling in C3H/HeJ and C57BL/10ScCr mice: mutations in Tlr 4 gene. *Science*.

[23] Hayashi F., Smith K. D., Ozinsky A. (2001). The innate immune response to bacterial flagellin is mediated by toll-like receptor 5. *Nature*.

[24] Lee S. K., Stack A., Katzowitsch E., Aizawa S. I., Suerbaum S., Josenhans C. (2003). Helicobacter pylori flagellins have very low intrinsic activity to stimulate human gastric epithelial cells via TLR5. *Microbes and Infection*.

[25] Hemmi H., Takeuchi O., Kawai T. (2000). A toll-like receptor recognizes bacterial DNA. *Nature*.

[26] Varga M. G., Shaffer C. L., Sierra J. C. (2016). Pathogenic Helicobacter pylori strains translocate DNA and activate TLR9 via the cancer-associated cag type IV secretion system. *Oncogene*.

[27] Ding S.-Z., Torok A. M., Smith M. F., Goldberg J. B. (2005). Toll-like receptor 2-mediated gene expression in epithelial cells during Helicobacter pylori infection. *Helicobacter*.

[28] Ihan A., Gubina M. (2014). The immune response to Helicobacter pylori. *Food Technology and Biotechnology*.

[29] Smith S. M., Moran A. P., Duggan S. P. (2011). Tribbles 3: a novel regulator of TLR2-mediated signaling in response to Helicobacter pylori lipopolysaccharide. *Journal of Immunology*.

[30] Schmausser B., Andrulis M., Endrich S., Müller-Hermelink H.-K., Eck M. (2005). Toll-like receptors TLR4, TLR5 and TLR9 on gastric carcinoma cells: an implication for interaction with Helicobacter pylori. *International Journal of Medical Microbiology*.

[31] Yokota S.-I., Okabayashi T., Rehli M., Fujii N., Amano K.-I. (2010). *Helicobacter pylori* lipopolysaccharides upregulate toll-like receptor 4 expression and proliferation of gastric epithelial cells via the MEK1/2-ERK1/2 mitogen-activated protein kinase pathway. *Infection and Immunity*.

[32] Takeda K., Akira S. (2005). Toll-like receptors in innate immunity. *International Immunology*.

[33] Yokota S., Ohnishi T., Muroi M., Tanamoto K., Fujii N., Amano K. (2007). Highly-purified Helicobacter pylori LPS preparations induce weak inflammatory reactions and utilize toll-like receptor 2 complex but not toll-like receptor 4 complex. *FEMS Immunology and Medical Microbiology*.

[34] Lepper P. M., Triantafilou M., Schumann C., Schneider E. M., Triantafilou K. (2005). Lipopolysaccharides from Helicobacter pylori can act as antagonists for toll-like receptor 4. *Cellular Microbiology*.

[35] Triantafilou M., Gamper F. G. J., Lepper P. M. (2007). Lipopolysaccharides from atherosclerosis-associated bacteria antagonize TLR4, induce formation of TLR2/1/CD36 complexes in lipid rafts and trigger TLR2-induced inflammatory responses in human vascular endothelial cells. *Cellular Microbiology*.

[36] Smith M. F., Mitchell A., Li G. (2003). Toll-like receptor (TLR) 2 and TLR5, but not TLR4, are required for *Helicobacter pylori*-induced NF-*κ*B activation and chemokine expression by epithelial cells. *Journal of Biological Chemistry*.

[37] Uno K., Kato K., Atsumi T. (2007). Toll-like receptor (TLR) 2 induced through TLR4 signaling initiated by Helicobacter pylori cooperatively amplifies iNOS induction in gastric epithelial cells. *American Journal of Physiology. Gastrointestinal and Liver Physiology*.

[38] Chochi K., Ichikura T., Kinoshita M. (2008). Helicobacter pylori augments growth of gastric cancers via the lipopolysaccharide-toll-like receptor 4 pathway whereas its lipopolysaccharide attenuates antitumor activities of human mononuclear cells. *Clinical Cancer Research*.

[39] Ishihara S., Rumi M. A. K., Kadowaki Y. (2004). Essential role of MD-2 in TLR4-dependent signaling during Helicobacter pylori-associated gastritis. *Journal of Immunology*.

[40] Kawahara T., Teshima S., Oka A., Sugiyama T., Kishi K., Rokutan K. (2001). Type I Helicobacter pylori lipopolysaccharide stimulates toll-like receptor 4 and activates mitogen oxidase 1 in gastric pit cells. *Infection and Immunity*.

[41] Bäckhed F., Rokbi B., Torstensson E. (2003). Gastric mucosal recognition of Helicobacter pylori is independent of toll-like receptor 4. *The Journal of Infectious Diseases*.

[42] Grimm M., Munz A., Exarchou A., Polligkeit J., Reinert S. (2014). Immunohistochemical detection of Helicobacter pylori without association of TLR5 expression in oral squamous cell carcinoma. *Journal of Oral Pathology & Medicine*.

[43] Khakzad M. R., Saffari A., Mohamadpour N. (2014). TLR4 and TLR2 expression in biopsy specimens from antral and corporal stomach zones in Helicobacter pylori infections. *Reports of Biochemistry and Molecular Biology.*.

[44] Amedei A., Cappon A., Codolo G. (2006). The neutrophil-activating protein of Helicobacter pylori promotes Th1 immune responses. *The Journal of Clinical Investigation*.

[45] Zeng H.-M., Pan K.-F., Zhang Y. (2011). Genetic variants of toll-like receptor 2 and 5, Helicobacter pylori infection, and risk of gastric cancer and its precursors in a Chinese population. *Cancer Epidemiology, Biomarkers & Prevention*.

[46] Hold G. L., Rabkin C. S., Chow W.-H. (2007). A functional polymorphism of toll-like receptor 4 gene increases risk of gastric carcinoma and its precursors. *Gastroenterology*.

[47] Cheng P.-L., Eng H.-L., Chou M.-H., You H.-L., Lin T.-M. (2007). Genetic polymorphisms of viral infection-associated toll-like receptors in Chinese population. *Translational Research*.

[48] Hishida A., Matsuo K., Goto Y. (2009). *Toll-like receptor* 4 + 3725 G/C polymorphism, *Helicobacter pylori* seropositivity, and the risk of gastric atrophy and gastric cancer in Japanese. *Helicobacter*.

[49] El-Omar E. M., Ng M. T., Hold G. L. (2008). Polymorphisms in toll-like receptor genes and risk of cancer. *Oncogene*.

[50] Merx S., Neumaier M., Wagner H., Kirschning C. J., Ahmad-Nejad P. (2007). Characterization and investigation of single nucleotide polymorphisms and a novel TLR2 mutation in the human TLR2 gene. *Human Molecular Genetics*.

[51] Smirnova I., Mann N., Dols A. (2003). Assay of locus-specific genetic load implicates rare toll-like receptor 4 mutations in meningococcal susceptibility. *Proceedings of the National Academy of Sciences of the United States of America*.

[52] Habibzadeh M., Tourani M., Shokri-Shirvani J., Mostafazadeh A., Khafri S., Nouri H. R. (2018). TLR2 Arg677Trp but not TLR2 -196 to -174 ins/del and Arg753Gln polymorphism alter the risk of peptic ulcer in north of Iran. *Journal of the Chinese Medical Association*.

[53] Echizen K., Hirose O., Maeda Y., Oshima M. (2016). Inflammation in gastric cancer: interplay of the COX-2/prostaglandin E2 and toll-like receptor/MyD88 pathways. *Cancer Science*.

[54] Maeda Y., Echizen K., Oshima H. (2016). Myeloid differentiation factor 88 signaling in bone marrow-derived cells promotes gastric tumorigenesis by generation of inflammatory microenvironment. *Cancer Prevention Research (Philadelphia, Pa.)*.

[55] Tye H., Kennedy C. L., Najdovska M. (2012). STAT3-driven upregulation of TLR2 promotes gastric tumorigenesis independent of tumor inflammation. *Cancer Cell*.

[56] Uno K., Kato K., Shimosegawa T. (2014). Novel role of toll-like receptors in Helicobacter pylori - induced gastric malignancy. *World Journal of Gastroenterology*.

[57] Takenaka R., Yokota K., Ayada K. (2004). *Helicobacter pylori* heat-shock protein 60 induces inflammatory responses through the toll-like receptor-triggered pathway in cultured human gastric epithelial cells. *Microbiology*.

[58] Schmausser B., Andrulis M., Endrich S. (2004). Expression and subcellular distribution of toll-like receptors TLR4, TLR5 and TLR9 on the gastric epithelium in *Helicobacter pylori* infection. *Clinical & Experimental Immunology*.

[59] Skvarc M., Stubljar D., Kopitar A. N. (2013). Inhibition of cathepsin X enzyme influences the immune response of THP-1 cells and dendritic cells infected with Helicobacter pylori. *Radiology and Oncology*.

[60] King J. D., Kocíncová D., Westman E. L., Lam J. S. (2009). Review: lipopolysaccharide biosynthesis in Pseudomonas aeruginosa. *Innate Immunity*.

[61] Li H., Liao T., Debowski A. W. (2016). Lipopolysaccharide structure and biosynthesis in Helicobacter pylori. *Helicobacter*.

[62] Faber J., Meyer C. U., Gemmer C. (2006). Human toll-like receptor 4 mutations are associated with susceptibility to invasive meningococcal disease in infancy. *The Pediatric Infectious Disease Journal*.

[63] Child N. J. A., Yang I. A., Pulletz M. C. K. (2003). Polymorphisms in toll-like receptor 4 and the systemic inflammatory response syndrome. *Biochemical Society Transactions*.

[64] Lundgren A., Strömberg E., Sjöling Å. (2005). Mucosal *FOXP3*-expressing CD4^+^ CD25^high^ regulatory T cells in *Helicobacter pylori*-infected patients. *Infection and Immunity*.

[65] Gong Y., Tao L., Jing L. (2016). Association of TLR4 and Treg in Helicobacter pylori colonization and inflammation in mice. *PLoS One*.

[66] Garza-Gonzalez E., Bosques-Padilla F. J., Mendoza-Ibarra S. I., Flores-Gutierrez J. P., Maldonado-Garza H. J., Perez-Perez G. I. (2007). Assessment of the toll-like receptor 4 Asp299Gly, Thr399Ile and interleukin-8 -251 polymorphisms in the risk for the development of distal gastric cancer. *BMC Cancer*.

[67] Stubljar D., Jeverica S., Jukic T. (2015). The influence of cytokine gene polymorphisms on the risk of developing gastric cancer in patients with Helicobacter pylori infection. *Radiology and Oncology*.

[68] Castaño-Rodríguez N., Kaakoush N. O., Goh K.-L., Fock K. M., Mitchell H. M. (2013). The role of TLR2, TLR4 and CD14 genetic polymorphisms in gastric carcinogenesis: a case-control study and meta-analysis. *PLoS One*.

[69] Kupcinskas J., Wex T., Bornschein J. (2011). Lack of association between gene polymorphisms of angiotensin converting enzyme, Nod-like receptor 1, toll-like receptor 4, FAS/FASL and the presence of Helicobacter pylori-induced premalignant gastric lesions and gastric cancer in Caucasians. *BMC Medical Genetics*.

[70] Guo Q., Zhu J., Xia B. (2006). Polymorphism of *CD14* gene but not the mutation of *TLR4* gene is associated with colorectal cancer in Chinese patients. *Journal of Gastroenterology and Hepatology*.

[71] Yuan M., Xia J., Ma L., Xiao B., Yang Q. (2010). Lack of the toll-like receptor 4 gene polymorphisms Asp299Gly and Thr399ile in a Chinese population. *The International Journal of Neuroscience*.

[72] Perrais M., Rousseaux C., Ducourouble M.-P. (2014). Helicobacter pylori urease and flagellin alter mucin gene expression in human gastric cancer cells. *Gastric Cancer*.

[73] Gewirtz A. T., Yu Y., Krishna U. S., Israel D. A., Lyons S. L., Peek R. M. (2004). *Helicobacter pylori* flagellin evades toll-like receptor 5-mediated innate immunity. *The Journal of Infectious Diseases*.

[74] Torok A. M., Bouton A. H., Goldberg J. B. (2005). Helicobacter pylori induces interleukin-8 secretion by toll-like receptor 2- and toll-like receptor 5-dependent and -independent pathways. *Infection and Immunity*.

[75] Andersen-Nissen E., Smith K. D., Strobe K. L. (2005). Evasion of toll-like receptor 5 by flagellated bacteria. *Proceedings of the National Academy of Sciences of the United States of America*.

[76] Forstnerič V., Ivičak-Kocjan K., Plaper T., Jerala R., Benčina M. (2017). The role of the C-terminal D0 domain of flagellin in activation of toll like receptor 5. *PLoS Pathogens*.

[77] Ivičak-Kocjan K., Panter G., Benčina M., Jerala R. (2013). Determination of the physiological 2:2 TLR5:flagellin activation stoichiometry revealed by the activity of a fusion receptor. *Biochemical and Biophysical Research Communications*.

[78] Jerala R., Pirher N., Ivicak K. Chimeric flagellins for vaccine.

[79] Dhiman N., Ovsyannikova I. G., Vierkant R. A. (2008). Associations between SNPs in toll-like receptors and related intracellular signaling molecules and immune responses to measles vaccine: preliminary results. *Vaccine*.

[80] Kang W., Rathinavelu S., Samuelson L. C., Merchant J. L. (2005). Interferon gamma induction of gastric mucous neck cell hypertrophy. *Laboratory Investigation*.

[81] Xu T., Fu D., Ren Y. (2017). Genetic variations of TLR5 gene interacted with Helicobacter pylori infection among carcinogenesis of gastric cancer. *Oncotarget*.

[82] Hawn T. R., Wu H., Grossman J. M., Hahn B. H., Tsao B. P., Aderem A. (2005). A stop codon polymorphism of toll-like receptor 5 is associated with resistance to systemic lupus erythematosus. *Proceedings of the National Academy of Sciences of the United States of America*.

[83] Kumar Pachathundikandi S., Brandt S., Madassery J., Backert S. (2011). Induction of TLR-2 and TLR-5 expression by *Helicobacter pylori* switches *cag*PAI-dependent signalling leading to the secretion of IL-8 and TNF-*α*. *PLoS One*.

[84] Haas T., Metzger J., Schmitz F. (2008). The DNA sugar backbone 2’ deoxyribose determines toll-like receptor 9 activation. *Immunity*.

[85] Ohto U., Shibata T., Tanji H. (2015). Structural basis of CpG and inhibitory DNA recognition by toll-like receptor 9. *Nature*.

[86] Wagner H. (2008). The sweetness of the DNA backbone drives toll-like receptor 9. *Current Opinion in Immunology*.

[87] Rad R., Ballhorn W., Voland P. (2009). Extracellular and intracellular pattern recognition receptors cooperate in the recognition of Helicobacter pylori. *Gastroenterology*.

[88] Otani K., Tanigawa T., Watanabe T. (2012). Toll-like receptor 9 signaling has anti-inflammatory effects on the early phase of Helicobacter pylori-induced gastritis. *Biochemical and Biophysical Research Communications*.

[89] Wang T. R., Peng J. C., Qiao Y. Q. (2014). Helicobacter pylori regulates TLR4 and TLR9 during gastric carcinogenesis. *International Journal of Clinical and Experimental Pathology*.

[90] Hold G. L., Rabkin C. S., Gammon M. D. (2009). CD14-159C/T and TLR9-1237T/C polymorphisms are not associated with gastric cancer risk in Caucasian populations. *European Journal of Cancer Prevention*.

[91] Wang X., Xue L., Yang Y., Xu L., Zhang G. (2013). TLR9 promoter polymorphism is associated with both an increased susceptibility to gastric carcinoma and poor prognosis. *PLoS One*.

[92] Trejo-de la O. A., Torres J., Sánchez-Zauco N. (2015). Polymorphisms in *TLR9* but not in *TLR5* increase the risk for duodenal ulcer and alter cytokine expression in the gastric mucosa. *Innate Immunity*.

[93] Ng M. T. H., Van’t Hof R., Crockett J. C. (2010). Increase in NF-*κ*B binding affinity of the variant C allele of the toll-like receptor 9 −1237T/C polymorphism is associated with *Helicobacter pylori*-induced gastric disease. *Infection and Immunity*.

[94] Ng M. W., Lau C. S., Chan T. M., Wong W. H. S., Lau Y. L. (2005). Polymorphisms of the toll-like receptor 9 (TLR9) gene with systemic lupus erythematosus in Chinese. *Rheumatology*.

[95] Mărginean M. O., Mărginean C. O., Meliţ L. E., Voidăzan S., Moldovan V., Bănescu C. (2017). The impact of host’s genetic susceptibility on *Helicobacter pylori* infection in children. *Medicine*.

[96] Peng G., Guo Z., Kiniwa Y. (2005). Toll-like receptor 8-mediated reversal of CD4^+^ regulatory T cell function. *Science*.

[97] Ram M. R., Goh K. L., Leow A. H. R. (2015). Polymorphisms at locus 4p14 of toll-like receptors *TLR-1* and *TLR-10* confer susceptibility to gastric carcinoma in *Helicobacter pylori* infection. *PLoS One*.

[98] Colotta F., Allavena P., Sica A., Garlanda C., Mantovani A. (2009). Cancer-related inflammation, the seventh hallmark of cancer: links to genetic instability. *Carcinogenesis*.

[99] Oliveira-Nascimento L., Massari P., Wetzler L. M. (2012). The role of TLR2 in infection and immunity. *Frontiers in Immunology*.

[100] Nagashima H., Iwatani S., Cruz M. (2015). Toll-like receptor 10 in Helicobacter pylori infection. *The Journal of Infectious Diseases*.

[101] Kim J. S., Chung S. J., Choi Y. S. (2007). Helicobacter pylori eradication for low-grade gastric mucosa-associated lymphoid tissue lymphoma is more successful in inducing remission in distal compared to proximal disease. *British Journal of Cancer*.

[102] Hong S. S., Jung H.-Y., Choi K. D. (2006). A prospective analysis of low-grade gastric malt lymphoma after Helicobacter pylori eradication. *Helicobacter*.

[103] Zhu Y., Wang C., Huang J. (2007). The Helicobacter pylori virulence factor Cag A promotes Erk 1/2-mediated Bad phosphorylation in lymphocytes: a mechanism of Cag A-inhibited lymphocyte apoptosis. *Cellular Microbiology*.

[104] Codolo G., Munari F., Fassan M., de Bernard M. (2015). Evaluation of the efficacy of the *H. pylori* protein HP-NAP as a therapeutic tool for treatment of bladder cancer in an orthotopic murine model. *Journal of Visualized Experiments*.

[105] Tran A. X., Stead C. M., Trent M. S. (2005). Remodeling of Helicobacter pylori lipopolysaccharide. *Journal of Endotoxin Research*.

[106] Zhao Y., Yokota K., Ayada K. (2007). Helicobacter pylori heat-shock protein 60 induces interleukin-8 via a toll-like receptor (TLR)2 and mitogen-activated protein (MAP) kinase pathway in human monocytes. *Journal of Medical Microbiology*.

[107] Pathak S. K., Basu S., Bhattacharyya A. (2006). TLR4-dependent NF-*κ*B activation and mitogen- and stress-activated protein kinase 1-triggered phosphorylation events are central to *Helicobacter pylori* peptidyl prolyl cis-, *trans*-isomerase (HP0175)-mediated induction of IL-6 release from macrophages. *The Journal of Immunology*.

[108] Persing D. H., Coler R. N., Lacy M. J. (2002). Taking toll: lipid A mimetics as adjuvants and immunomodulators. *Trends in Microbiology*.

[109] Koch M., Meyer T. F., Moss S. F. (2013). Inflammation, immunity, vaccines for Helicobacter pylori infection. *Helicobacter*.

